# Study feasibility - visualising the operationalisation of studies from early conception stage

**DOI:** 10.1186/1745-6215-16-S2-P23

**Published:** 2015-11-16

**Authors:** Irena Zwierska, Sarah Bathers

**Affiliations:** 1 Arthritis Research UK Primary Care Centre, Primary Care Sciences, Keele University, Keele, UK

## Background

Operationalising research studies in a primary care setting can be challenging, requiring considerable forward planning and liaison with various Clinical Trials Unit (CTU) staff, the Comprehensive Research Network (CRN) and external collaborators to ensure studies are feasible and delivered within the duration of funding.

## Objective

To visualise from early conception stage the operationalisation of research studies in a primary care setting, in terms of study feasibility within the funding time-frame.

## Methods

Operational flowcharts illustrating all processes and liaisons that need to occur within the study, together with Gantt charts detailing all stages of set-up, the submission and gaining of approvals, patient recruitment and follow-up, as well as data cleaning and analysis, are utilised by Keele CTU to provide an aide memoire to the study protocol with regards to study feasibility.

## Results

The methods used ensure that all studies, both simple and complex, can be easily visualised from an early conception stage, thus allowing efficient use of all resources to secure the delivery of studies to time and target.

## Conclusions

Detailed Gantt charts and visual, simplified, yet immensely beneficial flowcharts, can be easily embedded during grant application writing to illustrate the feasibility of studies within funding duration. The time investment in designing such tools at this stage allow studies to be visualised from an operational perspective early on, which help to ensure that studies can be delivered to time and target, once funded. Such tools should be implemented more widely to facilitate the smooth running of primary care research.

